# A novel push–pull central-lever mechanism reduces peak forces and energy-cost compared to hand-rim wheelchair propulsion during a controlled lab-based experiment

**DOI:** 10.1186/s12984-022-01007-5

**Published:** 2022-03-18

**Authors:** Thomas A. le Rütte, Fransisca Trigo, Luca Bessems, Lucas H. V. van der Woude, Riemer J. K. Vegter

**Affiliations:** 1 Center for Human Movement Sciences, University of Groningen, University Medical Center Groningen, Groningen, The Netherlands; 2 Center for Rehabilitation, University of Groningen, University Medical Center Groningen, Groningen, The Netherlands; 3 Peter Harrison Centre for Disability Sport, School of Exercise, Sport & Health Sciences, Loughborough University, Loughborough, UK

**Keywords:** Lever-propelled wheelchair, Wheelchair biomechanics, Peak force, Physical strain

## Abstract

**Background:**

Hand-rim wheelchair propulsion is straining and mechanically inefficient, often leading to upper limb complaints. Previous push–pull lever propulsion mechanisms have shown to perform better or equal in efficiency and physiological strain. Propulsion biomechanics have not been evaluated thus far. A novel push–pull central-lever propulsion mechanism is compared to conventional hand-rim wheelchair propulsion, using both physiological and biomechanical outcomes under low-intensity steady-state conditions on a motor driven treadmill.

**Methods:**

In this 5 day (distributed over a maximum of 21 days) between-group experiment, 30 able-bodied novices performed 60 min (5 × 3 × 4 min) of practice in either the push–pull central lever wheelchair (n = 15) or the hand-rim wheelchair (n = 15). At the first and final sessions cardiopulmonary strain, propulsion kinematics and force production were determined in both instrumented propulsion mechanisms. Repeated measures ANOVA evaluated between (propulsion mechanism type), within (over practice) and interaction effects.

**Results:**

Over practice, both groups significantly improved on all outcome measures. After practice the peak forces during the push and pull phase of lever propulsion were considerably lower compared to those in the handrim push phase (42 ± 10 & 46 ± 10 vs 63 ± 21N). Concomitantly, energy expenditure was found to be lower as well (263 ± 45 vs 298 ± 59W), on the other hand gross mechanical efficiency (6.4 ± 1.5 vs 5.9 ± 1.3%), heart-rate (97 ± 10 vs 98 ± 10 bpm) and perceived exertion (9 ± 2 vs 10 ± 1) were not significantly different between modes.

**Conclusion:**

The current study shows the potential benefits of the newly designed push–pull central-lever propulsion mechanism over regular hand rim wheelchair propulsion. The much lower forces and energy expenditure might help to reduce the strain on the upper extremities and thus prevent the development of overuse injury. This proof of concept in a controlled laboratory experiment warrants continued experimental research in wheelchair-users during daily life.

## Introduction

Around 90% of manual wheelchairs users (MWUs) employ a hand-rim propelled wheelchairs (HRW) as primary mode of locomotion for daily living [1]. However, HRW propulsion is a straining and mechanically inefficient mode of transportation. The combination of daily hand-rim wheelchair propulsion and transfers into and out of the wheelchair are thought to be responsible for high upper-limb strains and consequent prevalence of musculoskeletal complaints, oftentimes leading to inactivity [2–5].

Conventional HRW propulsion has a relatively low gross mechanical efficiency (GME); GME varies somewhere between 3 and 12%, depending among others on environmental conditions, speed, power output, wheelchair design, wheeling experience and propulsion technique [6, 7]. Compared to regular cycling (18–23%) [8] and handcycling (8–20%) [9, 10], hand-rim propulsion is an inefficient mode of locomotion.

The hand-rim wheelchair propulsion cycle is bi-phasic: an active push phase (~ 30% cycle time) and an inactive recovery phase, necessitating a continuous coupling and de-coupling of the hands to the hand-rims. The low fraction of the time for power transfer creates peak levels of force and work [11]. Spatial orientation of the humerus and scapula during HRW propulsion as well as in transfers, increase the musculoskeletal strain and risk of shoulder complaints, especially when they are paired with continued high external loads in daily life (e.g. floor surface, slopes, curb negotiation) [12].

The abovementioned factors suggest that any improvements in the propulsion mechanism and interfacing, may aid in reducing upper-body strains and complaints, while eventually promoting a physically active lifestyle [1, 13–17]. Apart from handcycling [17], few alternative propulsion mechanisms successfully entered and stayed in the marketplace. With ups and downs, lever-propelled wheelchairs have been available over the past 70 years in several different lever propulsion designs. Although crank-lever mechanisms exist [16], the most common designs involve two different levers each propelling one of the rear wheels individually [13]. In such a design, a push to the levers is similar to a push to the hand-rim and the handles can be pulled back during the recovery phase of the propulsion cycle. When tested on physiological performances, these types of wheelchairs proved to be more energy-efficient and less straining than HRWs [14–16, 18].

While bi-lever propulsion systems have been experimentally evaluated [1, 13, 14, 18, 19], a bi-manually driven single push–pull lever propulsion mechanism (PPLM) has very scarcely been tested on its physiological and biomechanical merit. The only studies that featured a design with a single lever originated in the 1970s and 1980s [15, 20, 21]. These early crank lever designs were different from the current design, as they were connected to the rear wheels and their cycle frequency was directly fixed to the speed, compared to the freewheel of the current design.

**Fig. 1:**
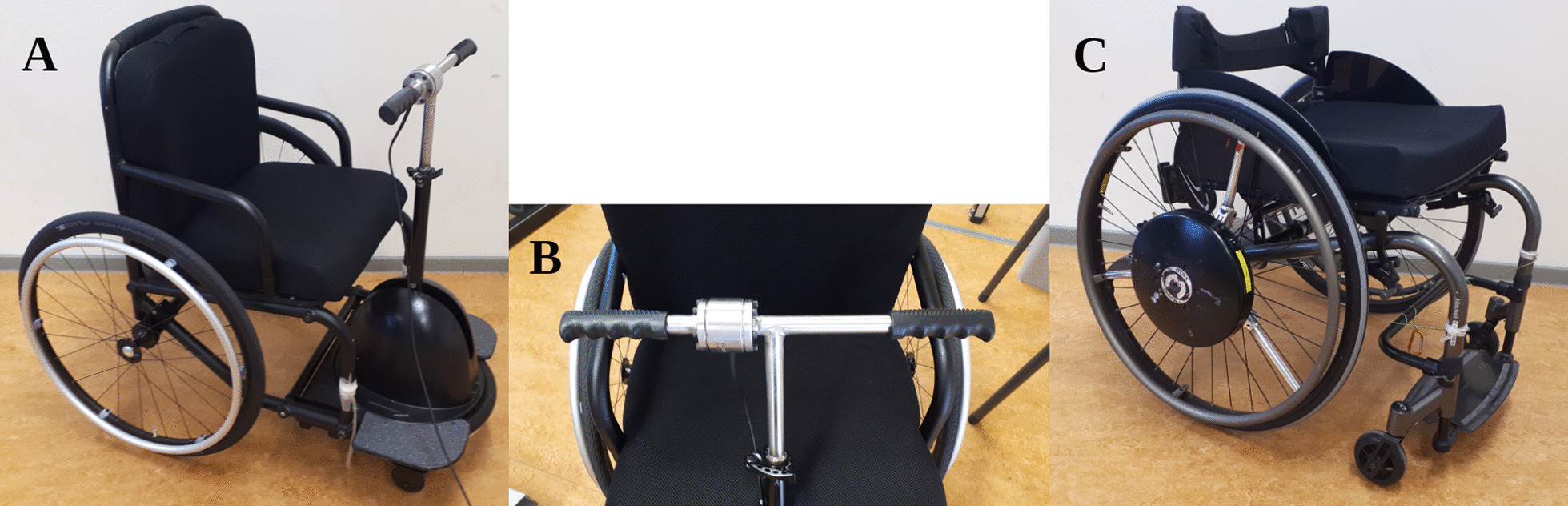
**A** The prototype of the push–pull single lever propulsion mechanism tested in the current study (RoChair; ROTA Mobility, California). **B** Close-up of the lever with the integrated unilateral force sensor at the right side. **C** The Küschall HRW with Smartwheel in the right rear wheel, which functioned as a control

The current study evaluates a new single central-lever push–pull design (rotamobility.com, Fig. 1A). This particular wheelchair design features a gearing system and a free-wheel, with which power can be transferred into forward motion in both the push and pull phases of the propulsion cycle, while it allows steering through the central lever mechanism. Utilisation of both phases of the cycle increases the portion of the propulsion cycle that can be used to exert force, thus theoretically reducing peak strain and potentially energy expenditure (EE) [22–24]. Moreover, the different directions of force application allow different muscle groups to be recruited. Therefore, the work is also divided over a larger total muscle mass, reducing strain on individual muscles.

Because of the novelty of the system, the current study does not yet compare experienced users of the PPLM to manual wheelchair users. Since early handrim wheelchair use is accompanied with motor learning processes [25, 26], it is assumed that the novel lever propulsion mechanism may require motor adaptation, skill acquisition and learning as well. Therefore, three research questions were addressed in this study. Firstly, does PPLM have physiological advantages over HRW, looking at EE, GME and HR? Secondly, are the peak forces needed to use an PPLM indeed lower than those needed for propelling a HRW? And finally, does practice change the outcomes of these questions with inexperienced users? These questions will be tested by comparing two inexperienced groups, either using the PPLM and a HRW before and after a practice intervention.

Based on previous studies on lever-propulsion [1, 13, 14, 18, 19], it was hypothesised that the PPLM will be less physiologically straining than a conventional HRW. This is expected to be shown in a higher GME and lower EE and HR. Also, forces that are applied on the lever of the PPLM are hypothesised to be lower than the forces applied on the handrim of the HRW, because the applied forces can be spread over a longer period of time of each full cycle and is divided in a push and pull action, involving different muscle sets. Therefore, equal power can be produced with lower peak forces.

These questions and hypotheses have been tested in a 5-day between-group experiment. In this experiment, 30 able-bodied novices performed 60 min of practice in either the push–pull central lever wheelchair (n = 15) or the hand-rim wheelchair (n = 15). At the first and final sessions cardiopulmonary stain, propulsion kinematics and force production were determined in both instrumented propulsion mechanisms.

## Methods

### Participants

For this study, 8 male and 22 female able-bodied novices volunteered. None of the participants had wheelchair experience before entering the study. Participants were informed about the experiment orally and in writing. All signed an informed consent before entering the study. A Physical Activity Readiness questionnaire (PAR-Q) was used to ensure safe study participation prior to experimentation. The research was approved by the ethical committee of the Centre for Human Movement Sciences Groningen, University Medical Centre Groningen (ECB/09.10.2012/1).

Participants were pseudo-randomly divided into two groups, the HRW group and the PPLM group, equally distributing male and female participants between the two groups. Also, because of the similarity of the lever propulsion motion to rowing, as mentioned by the developer of the RoChair, persons with rowing experience were equally split between the two groups. Participant characteristics are shown in Table 1.

**Table 1:** Participant characteristics (n = 2 × 15)

	Lever group	HRW group	
	Mean (SD)	Mean (SD)	t (p-value)
Gender	4 m/11f	4 m/11f	
Age (years)	21.3 (3.7)	20.9 (2.1)	0.36 (0.719)
Weight (kg)	71.8 (9.9)	73.4 (8.8)	0.47 (0.644)
Height (m)	1.74 (0.10)	1.79 (0.08)	1.51 (0.142)

### Experimental design

**Fig. 2:**
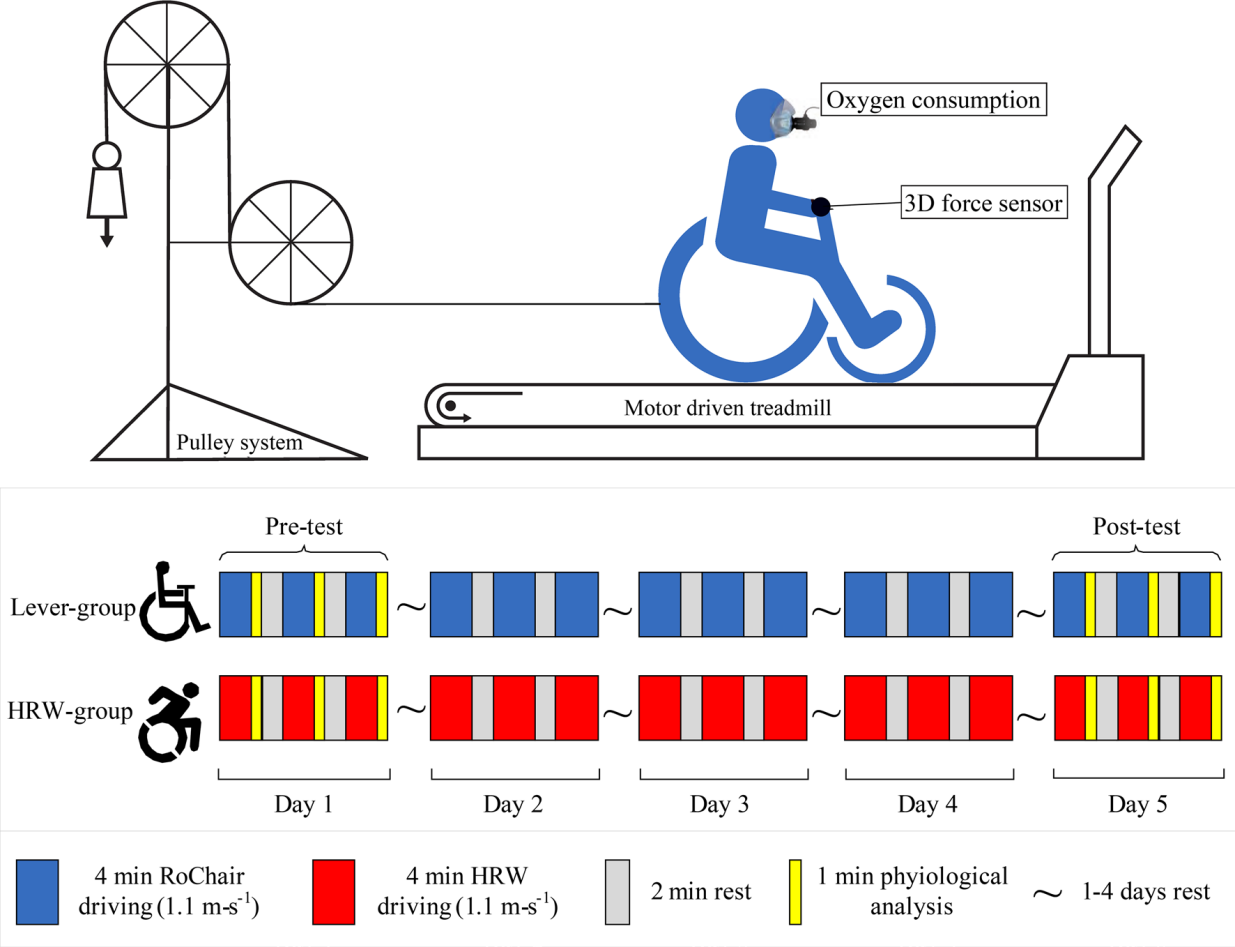
A schematic overview of the experimental setup (top) and experimental design (bottom) of this between-group (propulsion mode) five day (5 × 3 × 4 min) submaximal practice study

The lab-based experiment was performed on a level motorised treadmill (1.2 m wide, 2 m long; Motekforce Link, Amsterdam, the Netherlands, Fig. 2) at the Centre for Human Movement Sciences, University Medical Centre Groningen. After being divided into the two groups (Lever and HRW), the participants had a total of five 16 min sessions, on five non-consecutive days. Each day consisted of 3 × 4 min of practice in either the PPLM or the HRW. Prior to the first session, a drag test [21, 27] was performed to determine the rolling resistance of the wheelchair-participant combination. The external power output for both wheelchairs and during all conditions was held constant at 0.23 W/kg of bodyweight using a pulley system (27, see Fig. 2). The mass on the pulley system was calculated based on the rolling resistance from the individual drag test in each wheelchair-user combination and the—relative to individual body mass—determined external power output (0.23 W/kg) at the given belt speed. All five sessions consisted of three blocks of four minutes, in which the participants drove the wheelchair at a belt speed of 1.11 m∙s^−1^, with two minutes rest in between the blocks. After each block, participants gave an overall rating of perceived exertion (RPE) on a 15-point Borg scale [28]. An overview of the protocol is shown in Fig. 2. During the first and the last day of the protocol, physiological measures were determined, together with the kinetic properties of the propulsion. The total practice dose was 60 min.

### Instrumentation

During the pre and post sessions cardiopulmonary characteristics of the participants were measured breath-by-breath using the Quark CPET or the K5 (both COSMED, Rome, Italy), combined with a heart-rate monitor (Polar Electro, Hekele, Finland). Within participants, one of the two spirometers was used consistently over time. Additionally, equal numbers of participants were measured by either one spirometer in both groups to ensure no differences would be present as a result of different spirometer use.

### Wheelchairs

#### RoChair

The lever wheelchair that was tested was the first completed version of the RoChair (ROTA Mobility, Los Altos, CA, USA) (Fig. 1A). It features a propulsion mechanism in which positive power can be produced while both pulling and pushing. Similar to the gearing system of a bicycle, the propulsion mechanism included a freewheel, which enables the user to coast without moving the lever. In turn, this allowed the user to select their own preferred frequency and movement amplitude by pausing or returning motion at any given point. The lever was 0.57 m long with a 0.46 m wide handlebar. The handlebar was custom fitted with a K6D40 6-degree-of-freedom force/torque sensor (ME-Meßsysteme, Hennigsdorf, Germany) on the right hand side of the lever handlebar with a sample frequency of 100 Hz (Fig. 1B). The force sensor was calibrated to produce an output of 0 N when no force was applied on the handle and the lever was upright, having a 90˚ angle with the horizontal plane, measured with a digital inclinometer (Baseline, Fabrication Enterprises, White Plains, NY, USA). As the force sensor was incorporated into the handlebar, it rotated with the pivoting of the lever. The coordinate system of the force sensor rotated with it, therefore, the tangential force vector (perpendicular to the lever) was independent of the lever’s orientation. The total weight of the lever wheelchair was 24.7 kg, including the custom-made T-bar fitted with the force sensor. The rear wheels of the PPLM had a diameter of 0.66 m (26 inches) and the front wheel had a diameter of 0.32 m (12.5 inches). Tire pressure for all wheels was checked before measurements started, the rear wheels had a tire pressure between 600 and 700 kPa and the front wheel had a tire pressure of 240 kPa.

#### Hand-rim wheelchair

The HRW (Küschall, Witterswill, Switzerland; Fig. 1C) was fitted with an instrumented right rear wheel (Optipush, Max mobility, Lebanon, TN, USA; sample frequency = 200 Hz) and a weighted dummy on the left rear wheel. The total weight of the HRW including measurement wheel and dummy was 23.4 kg. The rear wheels of both wheelchairs had a diameter of 0.66 m (26 inches) and the front wheel of the RoChair had a diameter of 0.32 m (12.5 inches). Tire pressure for all wheels was checked before measurements started, the rear wheels had a tire pressure between 600 and 700 kPa and the front wheel of the RoChair had a tire pressure of 240 kPa.

### Data processing and analysis

All data were processed in MATLAB 2019a (Mathworks, Natick, MA, USA), using custom made algorithms [29]. From spirometer data of the pre and post sessions, only the fourth minute of each block was examined to ensure that a physiological steady-state was reached [30]. The full four minutes of data collected by other instruments were examined. In each block, the mean values measured during the relevant timeframe (the final minute for physiological data and four minutes for force and kinematic data) were determined. To produce a session value of each variable for one participant, the non-missing blocks were averaged into a single number.

Mean energy expenditure (EE) of the last minute was calculated from oxygen uptake (VO_2_, mL/min) and respiratory exchange rate (RER = VCO_2_/VO_2_) according to Eq. 1 [31]. GME was calculated using the experimentally set external power output (P_out_ (W)), which was kept 0.23 W/kg bodyweight with help of the drag test and the pulley system, and EE as shown in Eq. 2 [7].

1 $$EE \left(W\right)=\frac{\left(4.94\bullet RER+16.04\right)\bullet {\mathrm{VO}}_{2}}{60}$$

2 $$GME \left(\%\right)=\frac{{P}_{out}}{EE} \bullet 100\%$$

#### RoChair

**Fig. 3:**
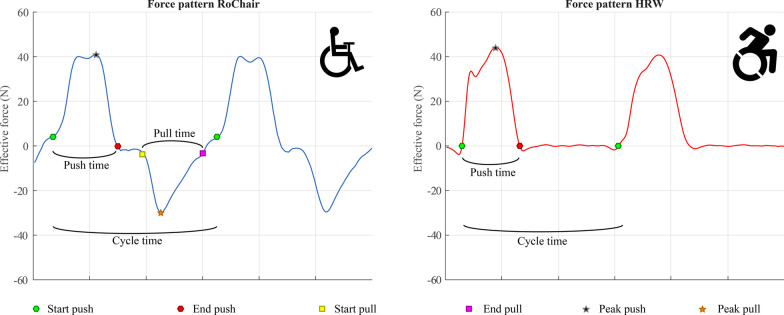
A typical example of the unilateral effective force patterns as measured in the force sensor of the RoChair (left panel) and the SmartWheel (right panel). Some key definitions in the data analysis are shown: push and pull phases for the lever propulsion cycle and the active push and non-active recovery phase of the full hand-rim propulsion cycle

**Fig. 4:**
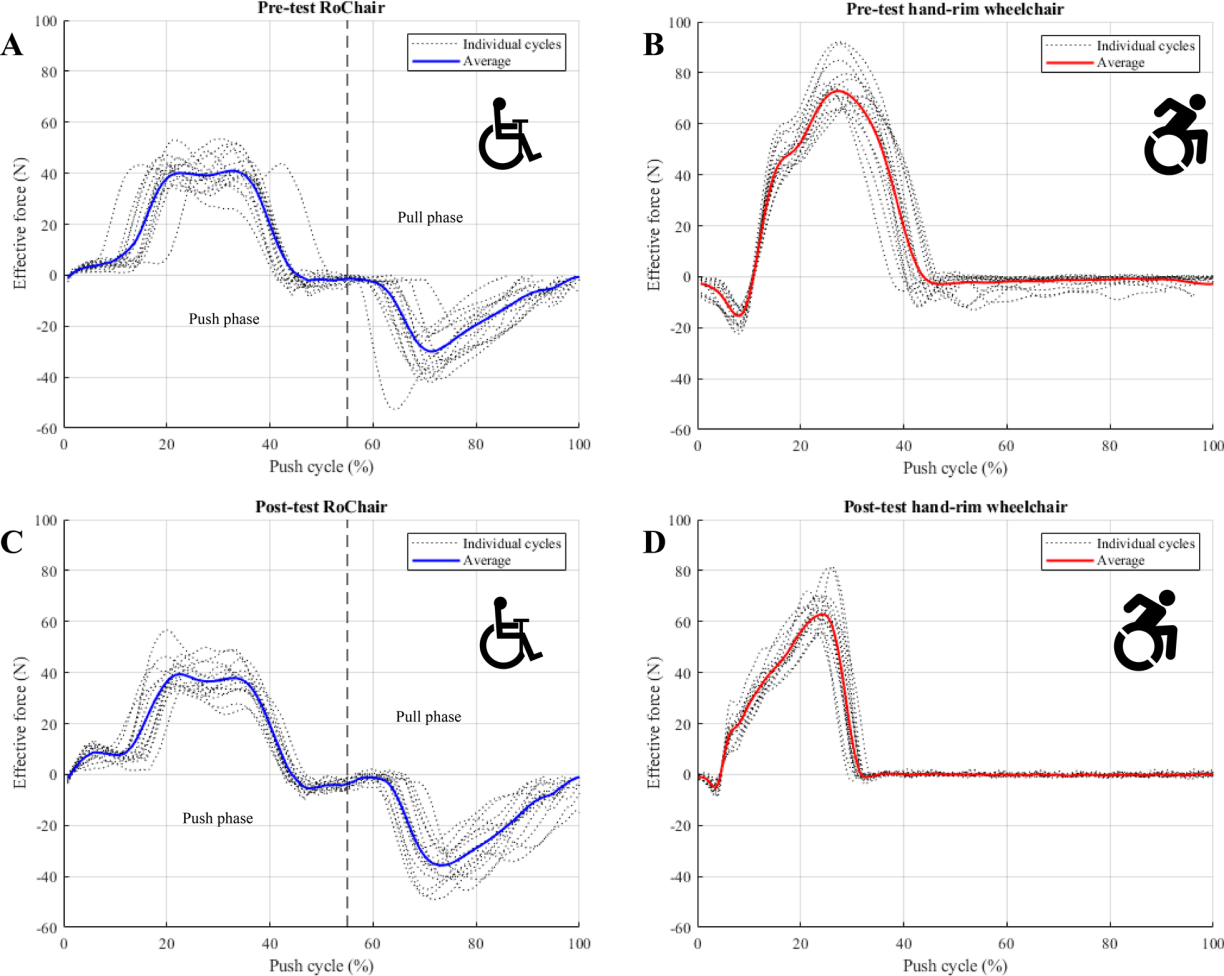
Effective propulsion forces for 15 cycles during a single block of two individual participants, one for the RoChair and one for the HRW. The blue line is the average of the 15 pictured cycles. **A** Pre-test in the RoChair. **B** Pre-test in the HRW. **C** Post-test in the RoChair. **D** Post-test in the HRW

From the force sensor data in the lever, individual cycles, and their push and pull phases, were identified as periods with a peak force over 10 N, positive for pushes and negative for pulls. Around 160 to 240 push cycles per four-minute block were identified, depending on the stroke frequency and angle that the participant selected. The start of a propulsion cycle was defined as the start of the push phase and the end of the cycle was the start of the next push phase. An example of the force patterns in two strokes is illustrated in Fig. 3, accompanied by the definitions described above. For Figs. 3 and 4, the effective force components were used, defined as the forces tangential to the rotation of the wheel or lever, creating forward movement. These components were used to give a clearer image of the pushing and pulling forces in the lever wheelchair. Per cycle, total cycle time, 3D peak pushing (F_push_) and pulling force (F_pull_) were calculated. The percentage of the cycle time spent actively on pushing or pulling the lever was calculated for the push phase and pull phase individually and added to make one active time percentage (%Active) that incorporated both active phases of force production in each cycle.

#### Hand-rim wheelchair

Similarly, individual cycles were defined from the Optipush data (Fig. 3), and cycle time, peak force (F_peak_), and %Active on the hand-rim were calculated. Cycle time was used to calculate SF. Peak push force and peak pull force were individually compared to the peak force of HRW propulsion for two main reasons: pushing and pulling forces differed substantially within certain participants and these differences were not homogenous between the participants.

### Statistics

Statistical analyses were performed using IBM SPSS Statistics 25 (IBM, Armonk, NY, USA). Participant characteristics and theoretical P_out_ of the groups were compared with an independent t-test. To compare the two propulsion modes and to evaluate the effect of practice, a mixed design analysis of variance (ANOVA) was performed with group as between-subjects factor time as the within-subjects factor. All data were checked for the assumptions of parametric testing. Because of the hypothesised differences, one-tailed test results were used for the individual time and group effects. Two-tailed results were used for the interaction effects. To determine whether the participants used more force when pushing or pulling, a paired t-test was performed between the peak push and pull forces. For this test, two-tailed p-values were used. The level of significance (α) for all analyses was 0.05.

## Results

All participants completed the experiment successfully. Six emergency stops, equally divided between the two propulsion modes in the early stage of the experiment, were made to ensure the safety of the participants. Two of the emergency stops were made because the participant drove the wheelchair outside the safe boundaries of the treadmill. The remaining four stops served to prevent experimental wires from getting entangled in the wheels or other moving parts of the wheelchair. All participants were able to continue the study.

In three participants, the heart rate monitor malfunctioned during one of the sessions. For this reason, the HR-data of the lever group had n = 14 for the first session and n = 13 for the last, resulting in a n = 12 for the lever group for which both sessions had complete valid data. Due to an error in the K5, one participant’s data for EE and GME was missing for the post test, resulting in n = 14 for the lever group. In other occasions of corrupted data, the two remaining blocks of the session were averaged and tested. Total external P_out_ that was theoretically set for the participants (calculated from bodyweight) of the lever group (mean = 16.5 ± 2.28) and the HRW group (mean = 16.8 ± 2.40) did not differ significantly (t(28) = -0.452, p = 0.655). No notable deviations from the standardised power and speed requirements were seen. Descriptive statistics and the outcomes of the repeated measures ANOVA are displayed in Table 2.

**Table 2:** Descriptive statistics (mean, SD and n) and statistical test results of the mixed design ANOVA (F, df, p-value and partial η^2^) for peak forces during the push phase (F_push_) and pull phase of lever propulsion (F_pull_), HRW propulsion (F_peak_), heart rate (HR), gross mechanical efficiency (GME), rating of perceived exertion (RPE), stroke frequency (SF) and active time percentage (%Active) of the hand-rim group and the lever group

			*Pre*	*Post*	*Propulsion modes*			*Practice*			*Interaction*		
Outcome	Propulsion mode	n	Mean (SD)	Mean (SD)	F (df)	p^†^	Partial η^2^	F (df)	p^†^	Partial η^2^	F (df)	p^††^	Partial η^2^
Fpush (N)	PPLM	15	45 (10)	42 (10)	24.91 (1.28)	< 0.001*	0.471	3.936 (1.28)	0.024*	0.123	1.341 (1.28)	0.257	0.046
Fpull (N)	PPLM	15	48 (15)	46 (10)	16.07 (1.28)	< 0.001*	0.365	3.160 (1.28)	0.043*	0.101	0.932 (1.28)	0.343	0.032
Fpeak (N)	HRW	15	71 (15)	63 (21)									
EE (W)	PPLM	14	283 (31.5)	263 (45)	7.428 (1.27)	0.006*	0.210	5.912 (1.27)	0.011*	0.174	0.221 (1.27)	0.642	0.008
	HRW	15	328 (54.7)	298 (59)									
GME (%)	PPLM	14	5.9 (0.91)	6.4 (1.46)	2.401 (1.27)	0.056	0.079	5.495 (1.27)	0.013*	0.164	0.003 (1.27)	0.959	< 0.001
	HRW	15	5.3 (1.06)	5.9 (1.33)									
HR (BPM)	PPLM	12	104 (17.4)	97 (10)	0.263 (1.25)	0.307	0.008	12.27 (1.25)	0.002*	0.300	0.474 (1.25)	0.497	0.029
	HRW	15	108 (19.9)	98 (10)									
SF (1/min)	PPLM	15	52 (11.8)	49 (6.0)	1.160 (1.27)	0.146	0.041	14.01 (1.27)	< 0.001*	0.342	4.947 (1.27)	0.035*	0.155
	HRW	14	63 (19.6)	50 (20.9)									
%Active	PPLM	15	75 (8.2)	86 (9.3)	597.6 (1.27)	< 0.001*	0.957	6.63 (1.28)	0.008*	0.197	12.53 (1.28)	0.001*	0.317
	HRW	14	33 (4.2)	31 (5.9)									
RPE (6–20)	PPLM	15	11.1 (2.5)	9.4 (1.7)	0.642 (1.28)	0.215	0.022	22.319 (1.28)	< 0.001*	0.444	0.002 (1.28)	0.963	< 0.001
	HRW	15	11.5 (2.0)	9.9 (1.3)									

^†^One-tailed p-value

^††^Two-tailed p-value

*Significant on an α = 0.05 level

Table 2 shows that the pre-post between-group experiment revealed significant effects between propulsion mechanisms. The experiment also shows overall significant practice effects for all of the analysed variables. Limited interaction effects indicate that the practice effects are similarly divided between the two propulsion modes.

### Lever vs. hand-rim

**Fig. 5:**
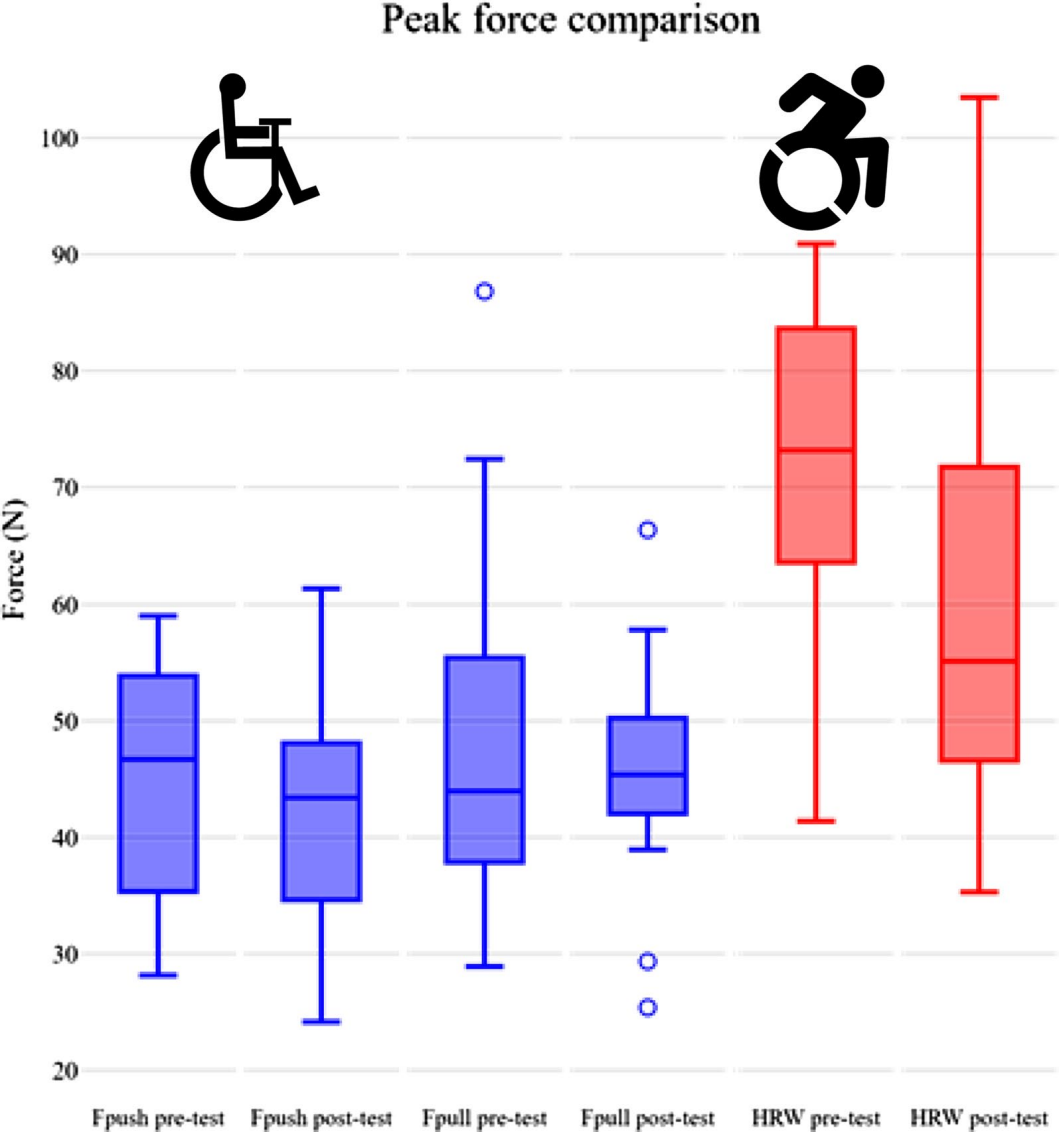
A boxplot displaying peak propulsion forces. The push and pull forces during lever propulsion (blue, n = 15) were approximately 33% lower than the forces during HRW propulsion (red, n = 15)

After practice, the peak push- (42 ± 10 N) and pull-forces (46 ± 10 N) during PPLM propulsion were significantly lower than the peak push force of HRW (63 ± 21 N) (Table 2, Fig. 4). Similarly, the energy expenditure of PPLM propulsion (263 ± 45 W) was lower than HRW propulsion (298 ± 59 W). As expected, the active portion of the propulsion cycle was considerably higher for PPLM (86 ± 9%) compared to HRW (31 ± 6). The effective force in Fig. 4 is the force that directly produces rotational torque in the lever or the wheels, also creating forward power for either wheelchair. While in HRW propulsion, a single peak is visible for each push cycle, two peaks are seen during lever propulsion with the RoChair, one for the push phase and a second downward peak for the pull phase, as shown in Fig. 4A and C. A summary of the propulsion forces is shown in Fig. 5. Apart from EE, no significant differences between the propulsion mechanisms were found for GME, HR, RPE and SF (Table 2).

### Practice effects

After practice, participants had significantly lower (peak) levels for F_push_, F_pull_, EE, HR, SF and RPE than at baseline (Table 2, Fig. 5). Also, a significant increase after practice was found for GME. The decrease of SF was larger in HRW propulsion than in lever propulsion, shown by the significant interaction effect (Table 2). Additionally, while there was an overall practice effect for %Active with an average increase for the total population, %Active increased for the PPLM group, but decreased for the HRW group, shown by the interaction effect.

## Discussion

The aim of this study was to compare the new push–pull lever mechanism (PPLM) to conventional hand-rim propulsion (HRW) during steady-state submaximal propulsion on a motor-driven treadmill in two groups of able-bodied novices over low-intensity lab-based practice. In line with our hypothesis, peak forces and energy expenditure were lower for PPLM, which held also after practice. Surprisingly the reduced energy expenditure for PPLM did not result in a higher mechanical efficiency for lever propulsion nor in differences among heart-rate nor perceived exertion. Self-selected push frequency did not differ between the two wheelchair types, but a larger portion of the propulsion cycle was effectively used in PPLM.

### Propulsion biomechanics

Peak forces during the push and pull phase of PPLM were around 33% (42 N vs 63 N) lower compared to the push phase of HRW propulsion. To our knowledge, there is no literature to compare those values and characteristics in lever propulsion. Two advantages of PPLM might explain the reduced peak forces during PPLM. First, the double propulsion phase of PPLM allowed force to be applied over a much longer period of the cycle, thus lowering the peak forces. Secondly, the moment arm of the lever (0.56 m) is larger than the radius of the rim (0.30 m), thus lower force is necessary to generate the same torque.

A force comparison can be made with handcycles, that to a certain extend share the same, bi-manual cyclic movement pattern. The peak forces per cycle that were found to be lower in the current study are similar to the results of a study that compared force application of handcycling to that of HRW propulsion [32]. Even though the propulsion pattern of the RoChair is not a completely continuous pattern as handcycling is, a much larger part of the cycle can be used for transmission of force than in a push cycle in HRW propulsion, as can be seen in Figs. 3 and 4. The PPLM used in the current study is aimed primarily on indoor use, which is why the comparison to a HRW was made. While the handcycle is more aimed for outdoor use, future studies comparing it to the PPLM could show interesting differences and similarities that could support or dispute any qualifications for the PPLM or handcycles.

### Push & pull phase

The pulling force is produced by different muscle groups than the pushing force and results in different loads on the joints. By all means, it employs theoretically a larger and more diverse muscle-mass distributed over the trunk, shoulders, elbows and wrists, additionally reducing muscle strain, while having lower activation peaks in individual muscles as represented in the considerably lower push and pull peak forces. Moreover, previous research on reverse wheelchair propulsion highlighted the benefits of utilising the larger muscle groups involved in the pulling motion in wheelchair use and on injury prevention [33, 34]. To learn more about how joint loads and muscle activity compares to hand-rim propulsion, future studies might focus on kinematic and electromyographic properties of RoChair lever propulsion. Subsequently, the potential of PPLM for prevention of overuse discomfort and injuries can be further substantiated and understood.

The orientation of the humerus and scapula during HRW propulsion is thought to be an important contributor to shoulder impingement in MWUs [12]. Because shoulder impingement has such high prevalence in MWUs, preventing it has high priority for wheeled mobility research in rehabilitation and adapted sports. Again similar to handcycling, lever propulsion is assumed more beneficial for shoulder and wrist positions—being in more natural joint positions- compared to hand-rim propulsion [35].

When comparing peak forces per cycle, no differences were found between pushing and pulling for both the pre- and the post-test. Nevertheless, concluding that all participants equally distributed pushing and pulling force would be wrong. High standard deviations, up to nearly a quarter of the means, illustrate that participants used multiple different strategies for force distribution between the two phases. Before this study, it was unknown how the PPLM would be used. Users could use the lever propulsion system similarly to a HRW and other lever systems, predominantly pushing the lever without strong pulling force, or choose to use the pulling function of the system and spread force out evenly between the two phases or even primarily use pulling force. The ability to choose a propulsion technique could be advantageous for potential users with limited trunk stability, as the lever can be pulled back with relative ease, while the backrest can stabilize when producing pushing force.

### Physiological properties

Energy expenditure during PPLM was lower compared to HRW, while maintaining the same power output. Remarkably, statistical analyses did not show a significant difference in GME, even though these measures are closely related to each other. It is, however, worth mentioning that the p-value for GME is very close to the alpha level of 0.05. Therefore, to fully dismiss the differences in GME found between the two wheelchairs would also not give an accurate representation. Possibly, the discrepancy between these related physiological measures can be explained by the imposed relative power relative to bodyweight. Because of this dependency on bodyweight, the distribution of GME on a group level is different to the group distribution for EE, which is seen in the statistical analyses. Illustratively, greater spread for GME than EE is visible in the data, as seen by consistently larger coefficient of variation (ratio between mean and SD).

In contrast with our hypothesis and most earlier research about physiological strain of lever propulsion [13, 14, 18, 19, 36], HR and RPE were not found to be different between the two propulsion modes. Yet, a performance decrement, as found by Zukowski and colleagues [37], using the RoChair is very unlikely, because of the already lower peak forces and energy expenditure for the current PPLM. Finding no difference in HR also means that the lever propulsion mechanism does not have worse physiological properties than HRW propulsion. This entails that exploring other differences between the two types of wheelchairs can give insight to possible advantages and disadvantages of an unconventional propulsion mechanism.

In line with previous research on early motor learning in wheelchair propulsion [25, 29, 38, 39], participants using the HRW improved their biomechanical performance over time. The same improvements were also seen for the participants after practice in the PPLM, indicating similarity in early motor learning between both modes. Therefore, results from earlier research on early motor learning with wheelchair propulsion may be translated into training strategies for PPLM motor learning.

### Comparison with previous lever designs

The design of the RoChair, one with a single lever positioned centrally in front of the user, is different from the designs that have been studied in the earlier researches. The gearing system that the lever is connected to ensures that both pushing and pulling force can be used to propel the wheelchair forward, this is not the case in the lever systems that were tested before. Secondly, the gearing system allows the user to select their own preferred frequency and movement amplitude by pausing or initiating the returning motion at any given point. This makes the design adaptable, because it facilitates more variety in propulsion acceleration, velocity and frequency of movement. Also, the system’s steering mechanism is straightforward, similar to a bicycle, and enables steering during coasting. The group effect shown for the active time percentage illustrated that the work can be spread over a larger portion of the cycle. While this is not directly shown to decrease physiological strain, it increases the smoothness of the propulsion which could reduce energy loss in acceleration and deceleration. However, results in the current study do not support this hypothesis. Data on control of the wheelchair and smoothness of the pace in the RoChair could provide more information on this relationship.

It is worth noting that more energy is likely to be lost in the chain drive mechanism of the RoChair, compared to other lever-wheelchair designs or HRW. Depending on the type of mechanism and the condition of the parts, efficiency of chain drive mechanisms can range between 65 and 98% [40]. Considering that the push–pull mechanism in the RoChair is fairly complicated and relatively low power is transferred, the efficiency of the mechanism is expected not to be in the highest range, even though the mechanism was in excellent condition. However, compensating for this energy loss may harm external validity and provide a flawed image of potential practical implication of the RoChair as a daily wheelchair, as it is an essential part of the propulsion mechanism.

### Study strengths and limitations and recommendations for future research

The current study was a highly standardised, highly controlled lab-based experiment. Controlling some of the many factors influencing wheelchair propulsion enabled a detailed biomechanical comparison.

The current study was conducted using able-bodied students as participants, this brings both strengths and limitations. Participants were equally untrained in both wheelchairs, therefore not favouring a propulsion mechanism. This allowed for equal practice routes and an unbiased look at initial motor learning. However, it does not accurately represent the population of MWUs. Trunk function, for example, can be severely affected in MWUs, yet is important in wheelchair propulsion [41]. Therefore, examining the contribution of the trunk in lever propulsion can provide information about the compatibility of lever wheelchairs with MWUs that experience weakened trunk function. However, the profile of the participants fit the aim of the study, to compare the mechanisms independently of users, very well.

The RoChair prototype that was used in the current study (Fig. 1A), still needs some modifications to be fit for use by most MWUs. The able-bodied participants experienced difficulty stepping into the wheelchair and getting up out of it. While this is not relevant yet when testing characteristics of the propulsion mechanism, this means that the model that was tested in the current study is not yet ready to be used by the intended population of MWUs. If the propulsion system could be attachable to the everyday wheelchair of MWUs, similarly to handcycle systems that are already available [42], this problem might be addressed. Additionally, pulling in a wheelchair has the risk of instability of the lower body and trunk in those with paralysis. It could be combined with a strap around the trunk or inclined seating and backrest, thus using gravity of the trunk and lower body as a counter weight for the pulling force.

Both wheelchairs were placed freely (no safety coupling to the frame of the treadmill, as is sometimes seen in wheelchair experiments) in the middle of the treadmill belt; in order to remain safely on the belt, participants themselves needed to actively control their coasting direction, going in a more or less straight line on the belt. Previous manual handrim studies have shown that this continuous control in combination with small steering errors leads to within-push differences on the left and right side that average out over the practice block and improve over practice [29, 43]. Possibly the lever-based simple steering mechanism of the PPLM may also have contributed to the reduced energy cost; clearly propulsion and steering are indeed coupled with this new system, as it is in handrim propulsion.

A drag test and a pulley-system were used to attain similar power output levels relative to a person’s body-weight for both modes. Thus, we could account for some of the wheelchair design properties and focus in the analyses as much on the propulsion principle itself. A final PPLM design must also be tested in real life to see whether the currently shown advantages outweigh possible increases in necessary power output at a certain speed due to weight and/or rolling and internal friction.

Future research should ideally also evaluate joint loads in the upper-limbs and shoulders, combined with the EMG measurement of muscle activity to further examine the positive effect of the PPLM, since this might provide critical information for developing propulsion mechanisms with lower injury prevalence. Full upper body kinematics, used as input for a musculoskeletal model could provide better insight into this [26]. Because MWUs body dynamics are assumed different to that of able-bodied people, analyses revolving around these dynamics should ideally be performed with the target population of MWUs. Studying MWU population, future research could also take into account wheelchair user experience, such as comfort and general usability. Also, analyses of power production and work per cycle would provide more insight in the biomechanics of lever wheelchair mechanisms. Finally, for stronger standardization, gear ratio and lever length were kept equal for the current study. However, exploring the possibility to vary both those parameters could reveal more efficient or comfortable propulsion.

## Conclusion

This first lab-based standardised treadmill experiment on the novel RoChair, a push–pull single central-lever propulsion mechanisms, showed a lower energy cost and push and pull peak forces compared to handrim wheelchair propulsion comparing two groups of novices following a low intensity practice period push–pull. Both lever propulsion and handrim wheeling showed a practice effect, indicating early motor learning in these cyclic tasks.

## Data Availability

The datasets used and/or analysed during the current study are available from the corresponding author on reasonable request.

## Abbreviations

%Active: Active percentage of the propulsion cycle
ANOVA: Analysis of variance
EE: Energy Expenditure
EMG: Electromyography
GME: Gross mechanical efficiency
HR: Heart rate
HRW: Hand-rim Wheelchair
MWU: Manual wheelchair user
PAR-Q: Physical activity readiness questionnaire
PPLM: Push–pull lever propulsion mechanism
RER: Respiratory exchange rate
RPE: Rate of perceived exertion
